# Self-Assembled Multivalent Aptamer Nanoparticles with Potential CAR-like Characteristics Could Activate T Cells and Inhibit Melanoma Growth

**DOI:** 10.1016/j.omto.2020.03.002

**Published:** 2020-03-13

**Authors:** Chenjun Bai, Shanshan Gao, Sai Hu, Xuemei Liu, Hui Li, Jie Dong, Aixue Huang, Lingling Zhu, Pingkun Zhou, Shaohua Li, Ningsheng Shao

**Affiliations:** 1 Institute of Military Cognition and Brain Sciences, Beijing 100850, China; 2 Institute of Radiation Medicine, Beijing 100850, China; 3 The Fifth Medical Center of PLA General Hospital, Beijing 100039, China

**Keywords:** multivalent, aptamers, self-assemble, T cells, melanoma

## Abstract

In this study, the CAR-like multivalent aptamer nanoparticles (X-polymers) were assembled with the dimer of murine CD28 RNA aptamer (CD28Apt7), the tetramer of CTLA-4 (cytotoxic T-lymphocyte-associated protein 4) RNA aptamer (Del60), and a folic acid labeled ssDNA fragment in a stable nucleic acid three-way junction scaffold (3WJ). Results showed that the X-polymers could recognize both the mCD28 and mCTLA-4 molecules. Confocal imaging and flow cytometry assays showed that the X-polymers could target both T cells and B16 cells *in vitro*. With the first costimulatory signals provided by the CD3 antibodies, the X-polymers could increase T cell proliferation and reverse the inhibitory effect of interleukin-2 (IL-2) secreting caused by exogenous B7.1 molecules on T cells *in vitro*. Results of our study also showed that X-polymers could inhibit mouse melanoma B16 cell growth both *in vitro* and *in vivo*. Our study demonstrated for the first time that the multivalent aptamer nanoparticle-activated T cells could fulfill the function of CAR-T, which promised a novel approach to developing a multi-functional design of aptamer drugs with potential CAR-like characteristics to enhance the safety of CAR-T cell immunotherapy.

## Graphical Abstract

**Figure:**
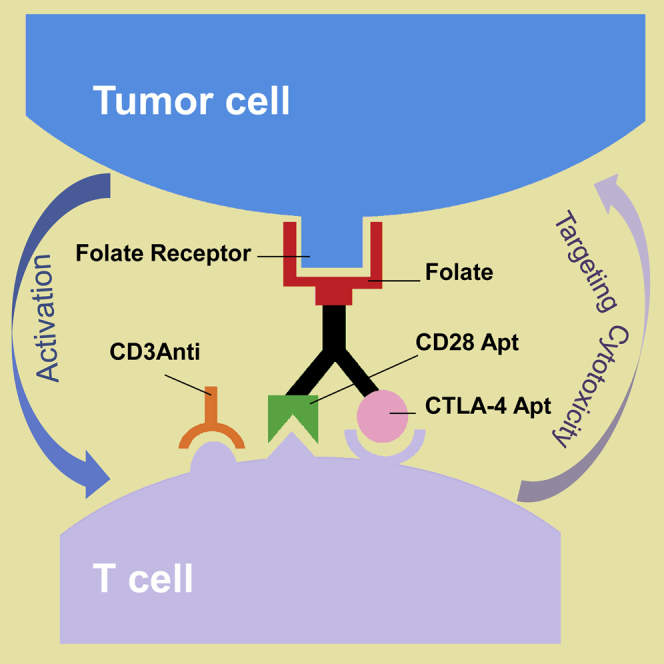

## Teaser

Shao and colleagues assembled multivalent aptamer nanoparticles with potential CAR-like characteristics. The CAR-like nanoparticles could activate T cells and inhibit melanoma growth both *in vitro* and *in vivo*. They put forward a concept of CAR-like aptamer drugs that may avoid the complexity of CAR-T preparation of exogenous transduction.

## Introduction

Tumor cells generated by genetic or epigenetic mutations express specific antigens, known as neo-antigens. These cells are usually monitored and eliminated once identified *in vivo* by the immune system. However, some tumor cells can evade the recognition of T cells, resulting in immune escape or tolerance. Immunotherapy of tumors refers to the restoration of the body’s anti-tumor immune response by means of the patient’s own immune response or drug assistance so that malignant tumor cells caused by gene mutation can be eliminated and immune surveillance can be performed.^1^ A number of immunotherapeutic strategies, including monoclonal antibodies, immune adjuvants, tumor vaccines, and CAR-Ts (chimeric antigen receptor T cells), have been widely used for the treatment of tumors so far.

Despite the varied causes of tumor immune escape, researchers have focused their attention on tumor immune escape caused by inhibited costimulatory molecules in recent years. Most of these costimulatory molecules belong to B7/CD28 immunoglobulin superfamily and tumor necrosis factor superfamily. Also, these molecules were named immune checkpoints as they are positively or negatively involved in the regulation of immunity.^2^ Immunotherapy of tumors based on immune checkpoints has developed rapidly in recent years. Monoclonal antibodies targeting CD28, CTLA-4, PD1, and PDL1 have been selling well on the market.^3^ More inspiringly, CAR-T, as a new tool based on immune checkpoint-modified T cells, has achieved remarkable results in the immunotherapy of tumors.

CAR-T refers to the use of chimeric antigen receptors to modify T cells so that T cells can play a more targeted and lethal role. The structure of CARs consists of an extracellular binding region, transmembrane region, and intracellular signal transduction region. What is critical about CARs is the extracellular recognition region for identifying tumors and generating activation signals and the intracellular signal transduction region for transducing stimulating proliferation signals to T cells, resulting in T cell proliferation and secreting cytotoxic factors. The most common extracellular recognition antigen is the CD19 molecule, which is used in the treatment of B cell malignant tumors by CAR-T.^4^^,^^5^ According to the different intracellular signal transduction regions, the first generation of the CAR system contains only one CD3ζ chain to transmit signals.^6^ On the basis of the first generation, the second- and third-generation CAR systems add one or two costimulatory signal units, such as CD28 or 4-1BB, to promote T cell proliferation, secrete costimulatory factors, and prolong the survival time of T cells.^7^^,^^8^

Compared with the aforementioned methods of tumor immunotherapy, CAR-T is flexible and changeable, and its strong adaptability to individualized treatment of cancer patients has come to the attention of not only scholars, but also biotechnology and pharmaceutical companies.^9^ Over the past two decades, CAR-T-related research has made breakthroughs in animal and clinical experiments and has shown good performance in the treatment of malignant tumors such as lymphoma^10^ and B cell leukemia.^11^^,^^12^ In August 2017, the first CAR-T drug, named Kymriah, was approved for marketing. Targeting CD19 to treat acute lymphoblastic leukemia (ALL) opened a new era of cell therapy.

The aptamers were first reported by Ellington and Szostak^13^ and Tuerk and Gold^14^ in 1990. The aptamer is a short single-strain nucleic acid (20- to 90-nt length) obtained by systematic evolution of ligands by exponential enrichment (SELEX) technology. It is a complex three-dimensional structure formed by interactions between single-stranded oligonucleotide bases and a specific binding of target substances, such as small molecules,^15^ proteins,^16^^,^^17^ living cells,^18^^,^^19^ and pathological sections.^20^ The aptamer has low immunogenicity and low molecular weight.^21^ When acting on the body as a drug or molecular delivery carrier, it will not produce few allergic reactions. As an alternative of antibody, it is widely used in diagnosis of diseases and drug development. For example, Han and Lee^22^ used RNA aptamers to rapidly diagnose *Salmonella typhimurium* infection. Liu et al.^23^ screened out an RNA aptamer binding to bovine thrombin, which could inhibit bovine coagulation activity. NX1838, as an aptamer for clinical treatment, can target vascular endothelial growth factors and has a good therapeutic effect against senile macular disease (ARMD).^24^ In recent years, the research of aptamers in the immunotherapy of tumors has also attracted much attention. The earliest aptamers related to immunity are the adaptors of CD4^+^ T cells.^25^ After a long period of research, these adaptors have proved to be able to inhibit virus replication and cytotoxicity.^26^ In 2003, Santulli-Marotto et al.^27^ demonstrated that the aptamer of CTLA-4 could be used in the immunotherapy of tumors. Since then, more immunotherapy-related aptamers have been reported, and the targets include CD28, OX40, PD1,^28^, ^29^, ^30^ VCAM1, P-selectin,^31^^,^^32^ and interferon (IFN)-gamma,^33^ transforming growth factor β (TGF-β),^34^^,^^35^ as well as other cytokines.

In this study, we intended to build up self-assembled multivalent CAR-like aptamer nanoparticles, which can activate T cells while targeting B16 mouse melanoma tumor cells. The CAR-like multivalent aptamer nanoparticles (X-polymers) were assembled with the dimer of murine CD28 RNA aptamer (CD28Apt7, published by Pastor et al.^28^) and the tetramer of CTLA-4 (cytotoxic T-lymphocyte-associated protein 4) RNA aptamer (Del60, published by Santulli-Marotto et al.^27^), as well as a folic acid labeled single stranded DNA (ssDNA) fragment in a stable nucleic acid three-way junction scaffold (3WJ).^36^ The aptamer CD28Apt7 was used for supplying the costimulatory signals to activate T cells, and the aptamer Del60 was used for immune checkpoint blockade. The folic-acid-labeled ssDNAs were used for targeting cancer cells. Our results showed that the X-polymers could target both T cells and B16 cells *in vitro*. With the CD3 antibodies providing the first costimulatory signals, the X-polymers could activate T cells to become CAR-like T, which could inhibit the melanoma growth *in vitro*. The results also showed that X-polymer nanoparticles could inhibit B16 cell growth *in vivo*. We demonstrated for the first time that the multivalent aptamer nanoparticle-activated T cells could fulfill the function of CAR-T. Our research can facilitate the development of a multi-functional design of aptamer drugs with potential CAR-like characteristics to improve the safety of CAR-T cell immunotherapy.

## Results

### Specific Binding of Aptamers to Their Target Proteins

**Figure 1:**
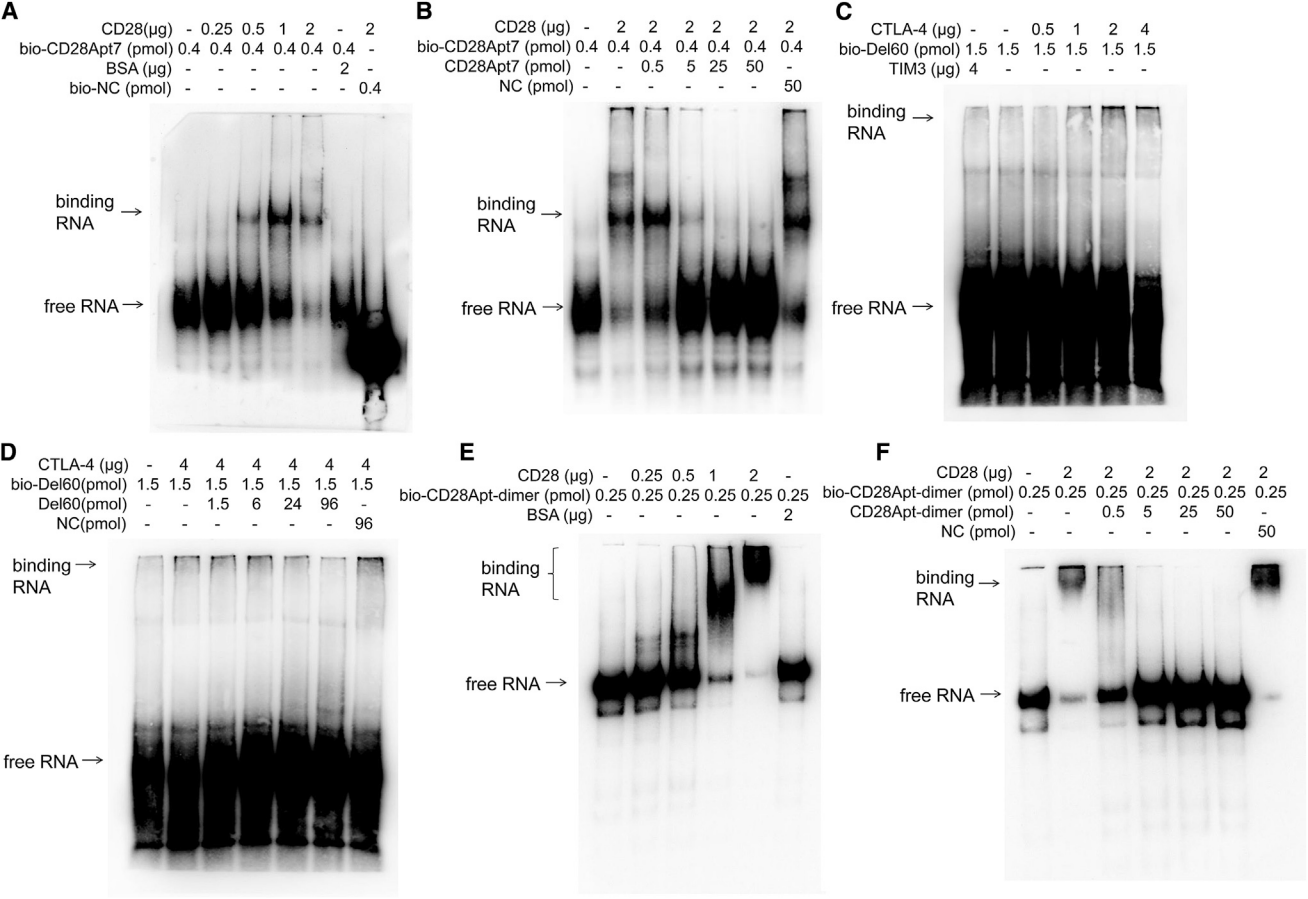
Verification of the Binding of Aptamers to Their Target Proteins Aptamers were modified with a 3′-terminal biotin modification kit, incubated with the target proteins in EP tube and analyzed by 8% or 10% native PAGE gel. (A and C) The binding of CD28Apt7 to CD28 protein (A) and Del60 to CTLA-4 protein (C). The more protein added, the more obvious the binding band was. BSA or TIM3 were used as the negative control target proteins. (B and D) The cold competitive binding results of the aptamers to CD28protein (B) and CTLA-4 protein (D), respectively. The biotin unlabeled aptamer was used to compete the biotin labeled aptamer to the same target protein. (E and F) The binding (E) and cold competitive binding (F) of the dimer of CD28Apt7 to CD28 proteins.

**Figure 2:**
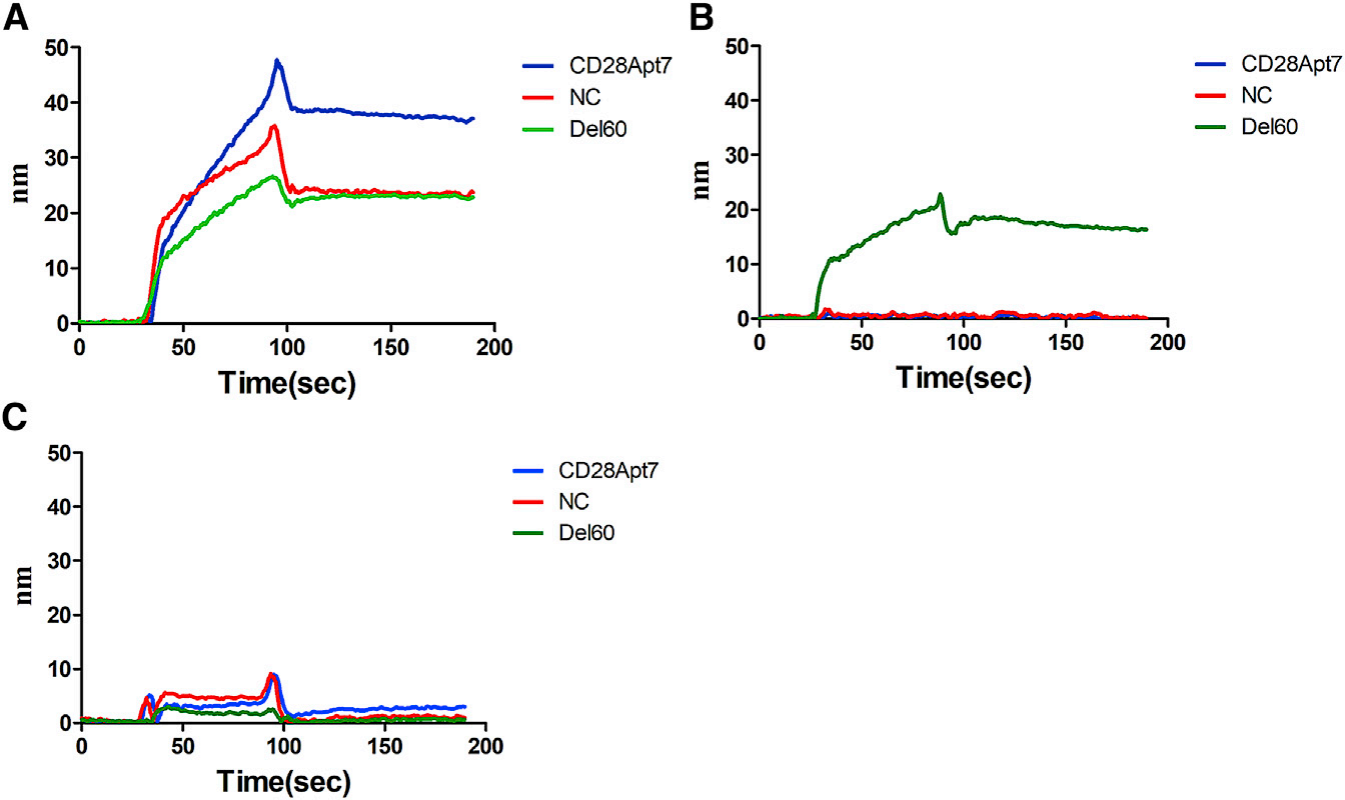
The Binding of Aptamers to Their Targets Confirmed with SPR Biotin-modified aptamers (200 nM) bind to streptavidin-labeled chips as solid phase, and target protein (1 μM) served as the mobile phase. The incubation buffer and cleaning buffer used was 20 mM HEPES, 150 mM NaCl, 2 mM CaCl2 (pH 7.4–7.6). (A) The binding of CD28Apt7 (blue), Del60 (green), and NC (red) to CD28 protein. (B) The binding of CD28Apt7 (blue), Del60 (green), and NC (red) to CTLA-4 protein. (C) The binding of CD28Apt7 (blue), Del60 (green), and NC (red) to a negative control protein (TIM3).

It is well known that T cells are activated through stimulation of the first and second signals before proliferation and differentiation. At the same time, immune checkpoint molecules such as PD1 and CTLA-4 are expressed on the surface of T cells exerting the negative feedback regulation, which will induce the immune escape of tumor cells when combined with ligands expressed on the surface of tumor cells. In order to effectively activate T cells, we used mouse CD28 RNA aptamer (CD28Apt7) as the activation factor and mouse CTLA-4 RNA aptamer (Del60) as the blocking factor of immunosuppressive pathway. We began by verifying the binding of aptamers to their target proteins by electrophoretic mobility shift assay (EMSA) and cold competitive binding assay. The results showed that CD28Apt7 and Del60 could specifically bind to CD28 and CTLA-4, respectively, in a concentration-dependent manner (Figures 1A–1D). According to the literature, the function of the CD28 aptamer in binding to CD28 molecules is in the form of dimer, so we verified the binding of the CD28 dimer to the CD28 protein by EMSA also. Results showed that the dimer of CD28Apt7 could also bind to mCD28 (Figures 1E and 1F). The binding of aptamers to their targets was also confirmed with a dual-channel surface plasmon resonance (Reichert4SPR) (Figure 2).

### Self-Assembly of X-polymer Nanoparticles and Verification of Their Binding to Target Proteins

**Figure 3:**
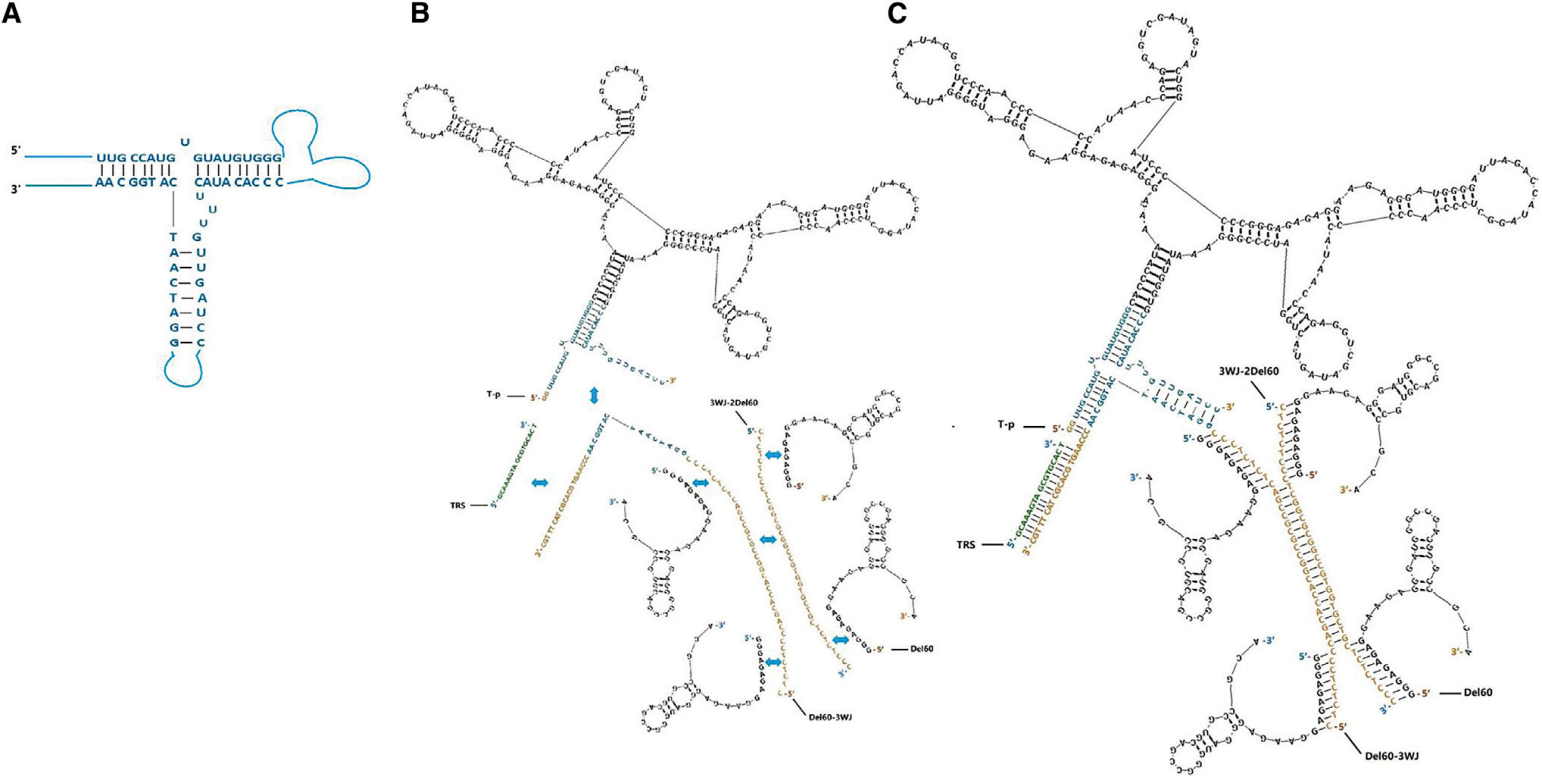
Constructs of X-polymer Nanoparticles (A) Construct of modified 3WJ structure. (B) Constructs of the T-p sequence composed of the 3WJ sequence (blue) and CD28Apt-dimer, the two skeleton sequences except the 3WJ sequence in the nonfunctional region (orange), the TRS sequence (green), and four Del60 aptamers. (C) Construct of self-assembled X-polymer nanoparticles. The X-polymer assembly consists of one CD28Apt-dimer and four Del60 aptamers. There are two base complementary regions of Del60 on 3WJ-2Del60 and Del60-3WJ, respectively. The secondary structure diagrams of CD28Apt-dimer aptamer and Del60 aptamer are drawn with RNA structure 5.7 software (black).

**Figure 4:**
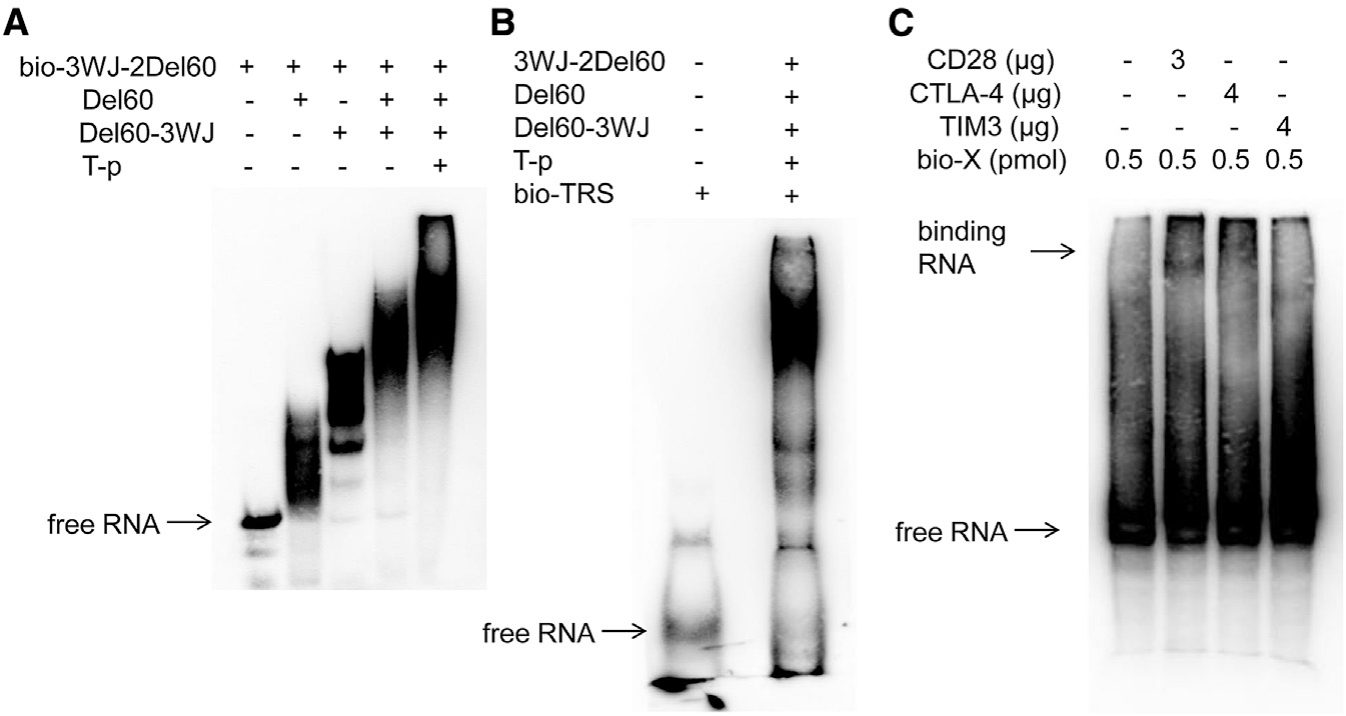
Self-Assembled X-polymer Nanoparticles and Verification of Their Binding to Target Proteins (A) Biotin-labeled 3WJ-2Del60 was assembled with Del60, Del60-3WJ, and T-p at the ratio of 1:4:1:1 (3WJ-2Del60:Del60:Del60-3WJ:T-p) and then identified by 10% native PAGE gel. (B) Biotin-labeled TRS was assembled with other components in X-polymer at the ratio of 1:4:1:1:1 (TRS:Del60:3WJ-2Del60:Del60-3WJ:T-p) and then identified by 10% native PAGE gel. (C) Self-assembled X-polymer nanoparticles with biotin-labeled TRS were identified to bind to CD28 and CTLA-4 in 8% native PAGE gel. The negative control target protein was TIM3.

X-polymer nanoparticles were constructed with a modified pRNA-3WJ central skeleton region and external aptamer functional region. The modified 3WJ structure was composed of two RNA sequences and one ssDNA sequence paired by complementary bases (Figure 3A). As shown in Figures 3B and 3C, the X-polymer nanoparticles were composed of one RNA sequence (named the T-p sequence), three ssDNA sequences (named Del60-3WJ, 3WJ-2Del60, and TRS, respectively), and four Del60 aptamers. In the T-P sequence, the CD28Apt7 dimer was inserted between two RNA fragments of the 3WJ structure. The Del60-3WJ sequence was the ssDNA sequence of 3WJ in which the 5′ ends were extended to complement to the folic-acid-modified TRS sequence and the 3′ ends were extended to the sequence complementary overlapping with two Del60 aptamers. 3WJ-2Del60 was a biotin-modified ssDNA sequence partially complementary to the Del60-3WJ sequence and overlapping with another two Del60 aptamers. EMSA results showed that the X-polymer nanoparticles were successfully self-assembled with the strands of Del60, 3wj-2Del60, Del60-3wj, TRS, and T-p at a molar concentration ratio of 4:1:1:1:1 in PBS buffer (Figures 4A and 4B) and could specifically bind to CD28 and CTLA-4 but not TIM3 (Figure 4C).

### X-polymer Nanoparticles Specifically Bind to Mouse T Cells and Tumor Cells

**Figure 5:**
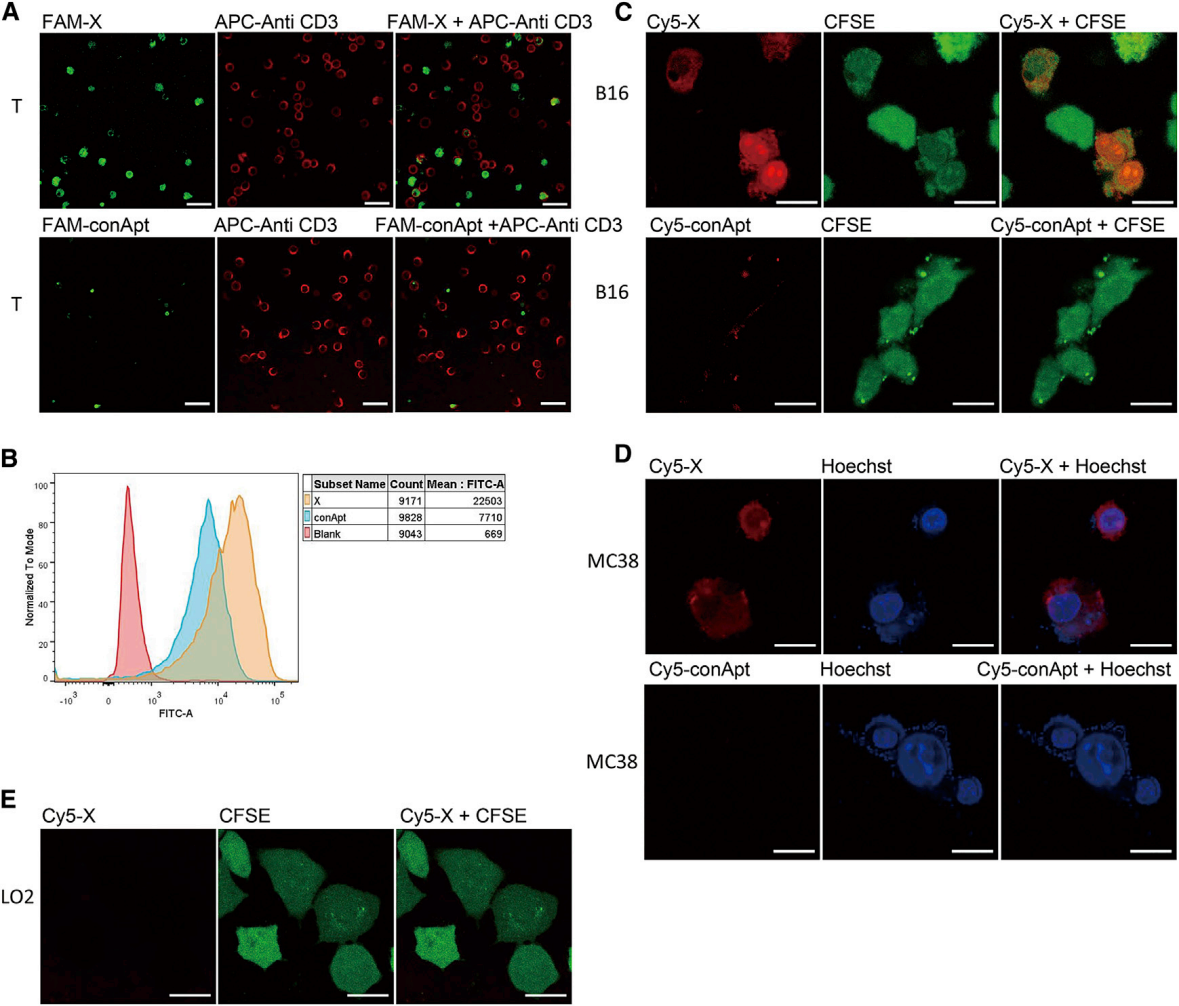
X-polymer Nanoparticles Specifically Bind to Mouse T Cells and Tumor Cells X-polymer nanoparticles (green fluorescence) were assembled with the FAM-labeled 3WJ-2Del60 sequence, and T cells were stained with anti-CD3 antibody (red fluorescence) labeled with APC. The co-localization of APC fluorescence and FAM fluorescence was observed under the confocal microscope (A). The binding of X-polymer nanoparticles to T cells was confirmed by flow cytometry as shown in (B). The Cy5-labeled 3WJ-2Del60 sequence was used to assemble X-polymer nanoparticles (red fluorescence). (C–E) The binding of X-polymer nanoparticles to B16 cells marked with CFSE (green fluorescence) (C), MC38 cells marked with Hoechst reagent (blue fluorescence) (D), and LO2 cells marked with CFSE (green fluorescence) (E) was observed under the confocal microscope. The ruler in the picture indicates a length of 20 μm.

T lymphocytes purified from the mouse spleen were recognized by allophycocyanin (APC)-labeled anti-CD3 antibody. The carboxyfluorescein (FAM)-labeled TRS ssDNA strand in X-polymer nanoparticles was used to indicate the binding of X-polymer nanoparticles to targeting cells. Confocal imaging showed that X-polymer nanoparticles could specifically bind to T cells (Figure 5A). The binding of X-polymer nanoparticles to T cells was also confirmed by flow cytometry assay (Figure 5B).

As epithelial-derived tumor cells highly express folate receptors, the folate labeled 5′ end of TRS in X-polymer nanoparticles may specifically bind to tumor cells expressing folate receptors. Our results showed that Cy5-labeled X-polymer nanoparticles could specifically bind to epithelial-derived mouse melanoma B16 cells (the results of folate receptor expression in B16 cells were as follows in Figure S1) and colon cancer MC38 cells rather than human normal hepatocyte L02 cells by confocal imaging (Figures 5C–5E). To find out whether the binding sites of X-polymer nanoparticles were on the surface of living cells, carboxyfluorescein succinimidyl amino ester (CFSE) (green fluorescence) was used to stain the cytoplasm of B16 cells and L02 cells, while Hoechst reagent (blue fluorescence) was used to indicate the nucleus of MC38 cells.

### X-polymer Nanoparticles Promote Mouse T Cell Proliferation and Blockade T Cell Immunosuppression *In Vitro*

The purified mouse T cells were labeled with CFSE and cultured on a 96-well plate with 1640 medium containing 10% fetal bovine serum. CFSE reagent labeling is one of the common methods to detect the proliferation of immune cells. CFSE reagent has no color and fluorescence until the esterase in the cell removes its acetate group and produces a carboxyl fluorescein succinimide ester that can emit bright fluorescence. Once T cells divide, CFSE fluorescence values are evenly distributed in two progeny cells to indicate cell proliferation and are detected by flow cytometry. For T cell proliferation, anti-mouse CD3 antibodies were used to provide the first signal, and X-polymer nanoparticles (with 2'-fluorine modification of U and C bases) or anti-CD28 antibodies were used to provide the second signal as a positive control.

**Figure 6:**
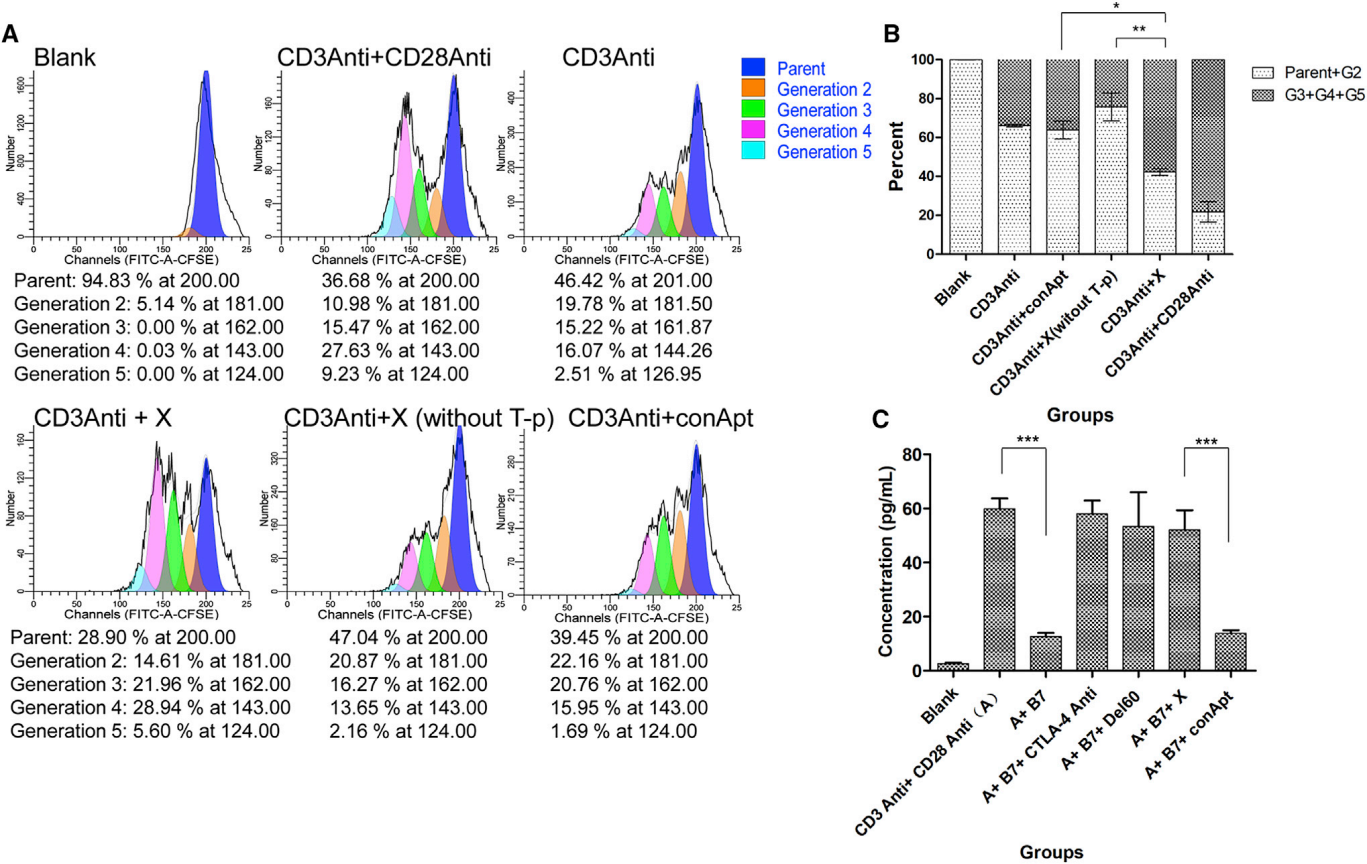
X-polymer Nanoparticles Promote Mouse T Cell Proliferation and Blockade T Cell Immunosuppression *In Vitro* (A) Flow cytometry analysis of the CD4^+^ T cells. T cells were labeled with CFSE; the first activation signal was provided by anti-CD3 antibodies and the second signal by anti-CD28 antibodies or X-polymer nanoparticles. T cell population was treated with PE-labeled anti-CD4 antibody and APC-labeled anti-CD8 antibody. (B) Histogram of the percentage of the CD4^+^ T cells analyzed by flow cytometry assay. (C) The content of IL-2 in T cell supernatant detected by ELISA. Blank refers to the group with only T cells and no antibodies or aptamer stimulation. The results are expressed as mean and SD of triplicates (∗p < 0.5, ∗∗p < 0.01, ∗∗∗p < 0.001).

The results showed that with the help of CD3 antibody stimulation, the X-polymer nanoparticles could significantly activate T cells and promote CD4^+^ T cell proliferation *in vitro*. Compared with the control aptamer (conApt), the percentage of parental T cells significantly decreased after 72 h of stimulation by X-polymer nanoparticles, which was similar to the result of CD28 antibody simulation. Results also showed that X-polymer without T-p sequence had no effect on the proliferation of T cells. There was significant difference between the groups of X-polymer with T-p and X-polymer without T-p for T cell proliferation (p < 0.001). It was proved that the effect of X-polymer on T cell proliferation was mainly induced by a T-p sequence that contained the dimer of CD28Apt7. There was no significant difference in T cell proliferation between the groups of X-polymer without T-p and conApt groups, indicating that the T cell stimulation in these two groups was mainly induced by CD3Anti antibodies (Figures 6A and 6B). However, the proliferation of CD8^+^ T cells was not obvious, because there was no antigen stimulation *in vitro* (Figure S2).

IL-2, as one of the important cytokines secreted by T cells, can help promote the growth and differentiation of T cells. The content of IL-2 in the cell supernatant could reflect the effect of the inhibitory pathway blocking. To test the extent to which the X-polymer nanoparticles or Del60 aptamer can inhibit T cell immunosuppressive pathway, IL-2 content in the cell supernatant was measured by ELISA. The results showed that the content of IL-2 in the T cell supernatant increased significantly after anti-CD3 antibodies and anti-CD28 antibodies were added but decreased after the addition of B7.1, a protein that shares the same ligand with CTLA-4 and CD28, which could inhibit the activation pathway of T cells by binding CTLA-4. X-polymer nanoparticles or Del60 aptamer alone could reverse the inhibiting effect of B7.1 by blocking the binding of B7.1 to CTLA-4, like anti-CTLA-4 antibodies (Figure 6C).

### X-polymer Nanoparticles Inhibit Mouse Melanoma B16 Cell Growth Both *In Vitro* and *In Vivo*

**Figure 7:**
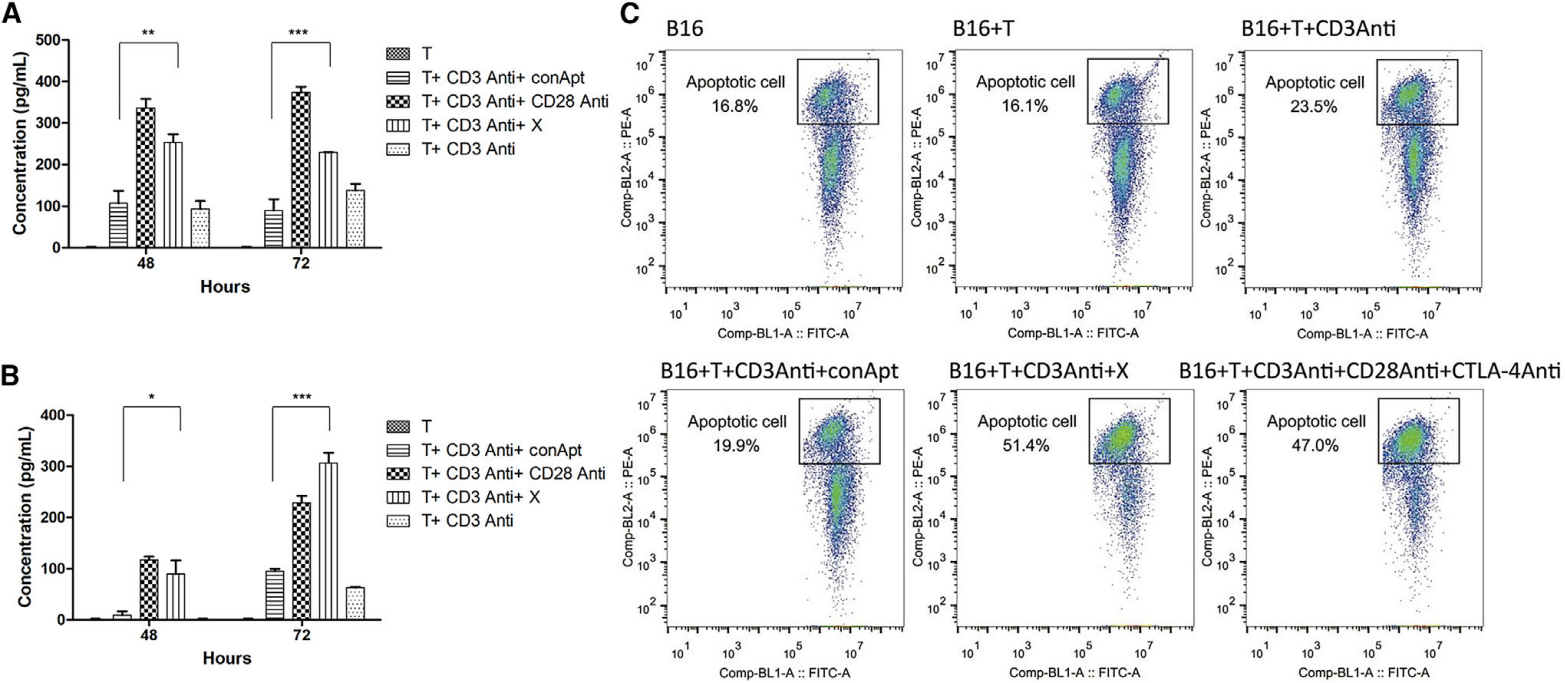
Measurement of the Secretion of IFN-γ and Granzyme B Levels in the Supernatant of Cultured T Cells and the Apoptosis Rate of B16 Cells Co-cultured with T Cells Activated with X-polymer Nanoparticles (A) The IFN-γ level in the supernatant of cultured T cells. (B) The granzyme B level in the supernatant of cultured T cells. (C) The apoptosis rate of B16 cells co-cultured with T cells. The horizontal axis of the coordinate is FITC (CFSE) fluorescence intensity, and the vertical axis of the coordinate is PE fluorescence intensity. The cells in the square frame were FITC + Annexin V + (the apoptotic B16 cells in all B16 cells).

Activated T cells could kill target cells by complicated means, such as by differentiating into cytotoxic T lymphocyte (CTL) cells, secreting perforin, granulase, interferon, and other factors, or activating the Fasl pathway. We detected the killing ability of activated T cells by measuring the secretion of IFN-γ and granzyme B. The results showed that the secretion of IFN-γ and granzyme B increased significantly after T cell activation stimulated by X-polymer nanoparticles compared with the control group (Figures 7A and 7B). To test their ability to directly kill tumor cells, X-polymer nanoparticle-activated T cells were co-incubated with melanoma B16 cells *in vitro*. Flow cytometry assay results showed that the apoptotic rate of B16 cells increased significantly, indicating that X-polymer nanoparticles could activate T cells that had enough cytotoxicity to target tumor cells (Figure 7C). At the same time, X-polymer nanoparticles alone did not cause the apoptosis of B16 cells (Figure S3).

**Figure 8:**
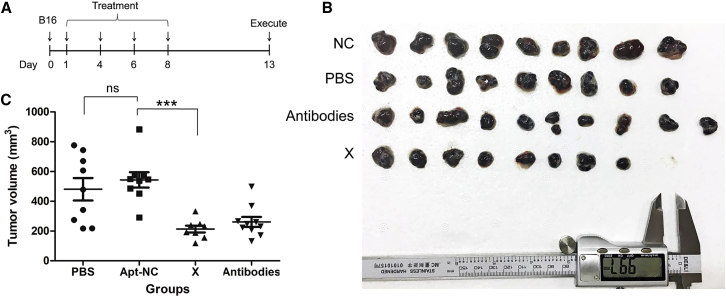
X-polymer Nanoparticles Inhibited Mouse Melanoma B16 Cell Growth *In Vivo* (A) Experimental outline of *in vivo* tumor model. B16 cells were injected into C57 mice by subcutaneous injection (number of mice in each group initially inoculated with tumor, n = 10), and animals were subsequently treated with either aptamers (X-polymer), antibodies (anti-CD28 antibodies plus anti-CTLA-4 antibodies), PBS, or negative control aptamers on days 1, 4, 6, and 8. (B) Measurement of tumor volume after removal of tumors (statistical number of mice in each group, NC, n = 9; PBS, n = 9; antibodies, n = 10; X, n = 8). As mentioned earlier, melanoma is a cystic tumor with soft texture. There are a lot of liquid tumor cells and necrotic cells in the capsule. In groups NC and PBS, one mouse was removed, respectively, because the tumor broke the skin, which led to the loss of tumor content and logistics and affected the accuracy of the experimental results. Some mice in group X did not grow tumors. It cannot be said whether the tumors were not inoculated or the drugs did not grow, so they were also removed from the group. (C) The results were analyzed by one-way ANOVA statistical method (ns, not significant, ∗∗∗p < 0.001).

We injected B16 mouse melanoma cells into C57 mice by subcutaneous injection and established a mouse tumor model. After the administration of X-polymer nanoparticles subcutaneously, tumor growth was significantly inhibited. X-polymer nanoparticles had a similar effect to anti-CD28 antibodies combined with anti-CTLA-4 antibodies (Figure 8). The results indicated that X-polymer nanoparticles could inhibit B16 cell growth *in vivo*.

## Discussion

The concept of self-assembled multivalent aptamer nanoparticles with potential CAR-like characteristics has been proposed for the first time in this study. Generally, any aptamers targeting immune checkpoints can be used for assembling multivalent aptamer nanoparticles, but what matters most is the binding activity of the aptamer to its target. To select the right aptamers, either the single-stranded DNA or RNA can be used, but assembled multivalent polymers whose components are all RNA molecules can be easily produced by direct transcription *in vitro*. In this paper, the CAR-like X-polymer nanoparticles were assembled with the dimer of CD28Apt7 and the tetramer of Del60, which could blockade the CD28 and CTLA-4, respectively. To prevent the formation of mismatched polymers, a stable intermediate 3WJ sequence was used as the skeleton structure.

As far as we know, the safety related to modifying and expanding CAR-T cells poses an obstacle to their clinical application. First, CAR-T therapy is an *in vitro* genetically modified cell therapy that involves taking T cells out of the patient blood, making autologous T cells express CARs through virus transduction and retransferring them back into the body for treatment. The process of preparing CAR-T is difficult due to the vulnerability to contamination.^37^ Second, according to many characteristics of host, tumor, and therapeutic agent, CAR-T therapy can induce inflammatory circuit, which can overwhelm the negative-feedback regulation mechanism of homeostasis and lead to cytokine storm.^38^ A few patients also had neurotoxic side effects, allergic reactions, and capillary leak syndrome (CLS). Kymriah, a product of CAR-T, was also questioned after it was launched in 2017.^39^ Although the side effects of CAR-T do not occur in every patient, these uncontrollable recurrences pose a threat to the life of patients. In addition, in the process of CAR-T treatment, the targeting recognition of cancer by CAR-T is also a key issue. Many immune-escaping cancer cells lack specific antigens, making it impossible to recognize and activate the CAR system accurately. And in the treatment of cancer with known targets, there is also a “miss-target effect” that is detrimental to normal tissues and organs of the body.^9^^,^^40^ Given these drawbacks of CAR-T therapy, the tedious and complicated preparation of CAR-T cells can be avoided, and the risk of introducing foreign pollutants when T cells are extracted or retransferred can be reduced if a chemical drug that activates T cells in the patient’s body can be developed.

Although the mechanism of cytokine storm is still controversial, it is likely that the over-activation of T cells or the over-release of cytokines caused by tumor cell death will lead to the inflammatory response of overload. It is the most urgent problem to be prevented or solved for tumor immunotherapy.^41^ We believe that on the basis of theory, the possibly mild T-cell-activating effect of CARs like aptamer drugs may reduce the cytokine burst-release syndrome, which needs to be confirmed in the future experiments.

The RNA molecules used in this paper were all modified with 2′-fluorine to prevent RNase degradation. The modified X-polymer nanoparticles may remain stable for a few days *in vivo*, which would provide mild stimulation of T cells. The controllable short time activation of T cells will put the CAR-like aptamer drugs at the advantage of decreasing the risk of the cytokine burst-release syndrome caused by over-activation of immune response. More importantly, an inadvertently triggered cytokine storm can also be brought under control by antagonists of aptamers. The simplest antagonists of aptamers are their reverse complementary RNA or ssDNA sequences.^23^^,^^42^ The aptamer will change from a single-chain structure to a double-chain structure when meeting with its reverse complementary sequence, losing its spatial folding ability and proper function eventually. This will make X-polymer nanoparticles safely controlled CAR-like drugs in future clinical applications.

There have been some studies on multifunctional aptamers, called bi-specific aptamers. Both the X-polymer nanoparticles and bi-specific aptamers consist of DNA or RNA aptamers. Bi-specific aptamers are generally composed of two aptamers, in which a simple random double-stranded DNA (dsDNA) sequence (or the same base A or T) forms a hinge connection.^43^^,^^44^ The X-polymer has the packaging RNA (pRNA)-like skeleton core sequence with strong binding force and stability. X-polymer targeted three molecular, two of which are surface molecules of T cells and one is surface molecule of tumor cells. The function of X-polymer is also different from that of bispecific aptamers. X-polymer combines with T cells and plays the role of killing tumors with the characteristics of T cells. It is similar to CAR-T in form, so we prefer to call it a CAR-like system.

As mentioned above, the X-polymer nanoparticles can be assembled with any costimulatory molecule or immune checkpoint aptamers needed. It is reported that the combination of immuno-checkpoint molecules that promote or inhibit the activation of T cells plays an important role in anti-tumor immunotherapy.^45^^,^^46^ Compared with the single signal activation mechanism of CAR system, the X-polymer nanoparticles with the dual stimulation signals for T cells had unique advantages. By increasing the number of immune checkpoint aptamers or by increasing the synergistic effect with costimulatory molecules, as with the third generation of CAR-T,^47^ the lethality to tumors can possibly be increased. In view of the diversity of aptamers and cell-surface binding sites, we can select specific binding aptamers for different tumor cells. Tumor-specific aptamers^48^ can be assembled on X-polymer instead of folate sequence to enhance the targeting of X-polymer and reduce the effect of tumor off-target.

In the experiment, we found that the aptamer control group had nonspecific binding with CD28 protein or T cells *in vitro*, but we confirmed that the control aptamers did not provide the second stimulation signal for T cells. We suspected that CD28 may probably be a nucleic acid binding protein, which also needs to be verified in future experiments.

In conclusion, we developed self-assembled CAR-like X-polymer nanoparticles that could activate T cells and effectively inhibit the growth of melanoma B16 cells both *in vitro* and *in vivo*. Our results point to a new direction in which to develop a multi-functional design of aptamer drugs with potential CAR-like characteristics to effectively address the safety concerns about CAR-T cell immunotherapy.

## Materials and Methods

### Cell Culture

The human immortalized normal hepatocyte cell line LO2 and cell line HepG2 were maintained in our lab. The mouse tumor cell lines B16 and MC38 were purchased from Hunan Fenghui Biotechnology. The cells were cultured in Dulbecco’s Modified Eagle’s Medium (DMEM) or RPMI 1640 medium (GIBCO, USA) supplemented with 10% fetal bovine serum (GIBCO) and antibiotics (100 μg/mL streptomycin and 100 U/mL penicillin, Sigma) in a humidified incubator containing 5% CO_2_ at 37°C.

### Preparation of Aptamers

The aptamer sequences, DNA templates, and primer sequences used in the experiments were as follows:

Aptamers and skeleton sequences were as follows:

**Del60-3wj**, 5′-CTCTCTCCCC AGCACCACGG CCGCGCCGAC TCTCTCCCGG ATCAATCATG GCAACCCAAG TGCACGCTAC TTTGC-3′,
**3WJ-2Del60**, 5′-CTCTCTCCCT CGGCGCGGCC GTGGTGCTGC TCTCTCCC-3′,
**T-p**, 5′-GGGCACCCAU AAAAGGGAGA GAGGAAGAGG GAUGGGGAUU AGACCAUAGG CUCCCAACCC CCAUAACCCA GAGGUCGAUA GUACUGGAUC CCCCCGGGAG AGAGGAAGAG GGAUGGGGAU UAGACCAUAG GCUCCCAACC CCCAUAACCC AGAGGUCGAU AGUACUGGAU CCCGGGAAAU AUGGGUGCCC ACAUACUUUG UUGAUCC-3′,
**DEL60**, 5′-GGGAGAGAGG AAGAGGGAUG GGCCGACGUG CCGCA-3′,
**TRS**, 5′-GCAAAGTAGC GTGCACT-3′,
**CD28Ap7**, 5′-GGGAGAGAGG AAGAGGGAUG GGGAUUAGAC CAUAGGCUCC CAACCCCCAU AACCCAGAGG UCGAUAGUAC UGGAUCC-3′,
**CD28Ap7-dime**r, 5′-GGGAGAGAGG AAGAGGGAUG GGGAUUAGAC CAUAGGCUCC CAACCCCCAU AACCCAGAGG UCGAUAGUAC UGGAUCCCCC CGGGAGAGAG GAAAGGGAU GGGGAUUAGA CCAUAGGCUC CCAACCCCCA UAACCCAGAG GUCGAUAGUA CUGGAUCCCC CC-3′,
**3WJ1**, 5′-UUGCCAUGUG UAUGUGGG-3′,
**3WJ2**, 5′-CCCACAUACU UUGUUGAUCC-3′,
**3WJ3**, 5′-GGATCAATCA TGGCAA-3′.

Double-stranded DNA templates and primers were as follows:

**T-p dsDNA template**, 5′-GAGTCTAATA CGACTCACTA TAGGGTTGCC ATGTGTATGT GGGCACCCAT AAAAGGGAGA GAGGAAGAGG GATGGGGATT AGACCATAGG CTCCCAACCC CCATAACCCA GAGGTCGATA GTACTGGATC CCCCCGGGAG AGAGGAAGAG GGATGGGGAT TAGACCATAG GCTCCCAACC CCCATAACCC AGAGGTCGAT AGTACTGGAT CCCGGGAAAT ATGGGTGCCC ACATACTTTG TTGATCC-3′,
**CD28Ap7-dimer dsDNA template**, 5′-GAGTCTAATA CGACTCACTA TAGGGAGAGA GGAAGAGGGA TGGGGATTAG ACCATAGGCT CCCAACCCCC ATAACCCAGA GGTCGATAGT ACTGGATCCC CCCGGGAGAG AGGAAGAGGG ATGGGGATTA GACCATAGGC TCCCAACCCC CATAACCCAG AGGTCGATAG TACTGGATCC CCCC-3′,
**XT7 F primer**, 5′-GAGTCTAATA CGACTCACTA TAGGG-3′,
**T-p R primer**, 5′-GGATCAACAA AGTATGTGGG C-3′,
**CD28Ap7 R primer**, 5′-GGGGGGATCC AGTACTATC-3′.

Del60, 3WJ1 and, 3WJ2 were synthesized and modified with with 2′-fluorine modification of U and C bases by Guangzhou RiboBio. Del60-3wj, 3WJ-2Del60, 3WJ3, and primers were synthesized by Sangon Biotech. TRS was synthesized and modified with 5′-folic acid by TAKARA. dsDNA templates were synthesized by TSINGKE Biological Technology. Fluorescence modification of all sequences was by Sangon Biotech. dsDNA templates of CD28Ap7 and CD28Ap7 dimer aptamers were obtained from PCR using XT7 F and CD28Ap7 R primers and purified after gel cutting. RNA aptamers were obtained from their dsDNA templates by a TranscriptAid T7 high-yield transcription kit (Thermo Scientific, K0441) *in vitro*. 2′-fluorine-modified aptamers for *in vitro* and *in vivo* experiments were obtained from their dsDNA templates by a DuraScribe T7 transcription kit (Lucigen, DS010925). The negative control RNA used for EMSA experiments was 3WJ1 (negative control, NC). The negative control fragments for *in vitro* and *in vivo* tumor cell experiments were assembled by 3WJ1, 3WJ2, and 3WJ3 at a ratio of 1:1:1 *in vitro* (conApt).

### EMSA

The EMSA experiment was conducted using a chemiluminescent nucleic acid detection module kit (Thermo Scientific Pierce, 89880). The Pierce RNA 3′ end biotinylation kit (Thermo, 20160) was used in the 3′ end biotin modification of the aptamer. Aptamer denaturation was as follows: RNA aptamers were placed at 85°C, 10 min (ssDNA fragment at 95°C, 10 min) and cooled on ice for 10 min. Target recombinant proteins (mouse CD28, Abcam, ab207143; mouse CTLA-4, Sino Biological, 50503-M08H; mouse PD1, Sino Biological, 50124-M08H; mouse TIM3, Sino Biological, 51152-M08H; mouse TIGIT, Sino Biological, 50939-M08H) and aptamers were incubated in buffer (20 mM HEPES, 150 mM NaCl, 2 mM CaCl_2_ [pH 7.4–7.6]]) at 37°C for 30– 60 min before being applied in native polyacrylamide gel electrophoresis. After being transferred to nylon membrane (GE Healthcare, RPN303B), the shift bands were UV cross-linked (1200 J energy, 254 nm wavelength) for 2 min and imaged using enhanced chemiluminescence (ECL) reagents with Clinx ChemiScope 3400 mini.

### Binding Analysis by SPR

The binding of the aptamers to their target proteins was analyzed by using Reichert4SPR. Biotin-modified aptamers (200 nM) bind to streptavidin-labeled chips as the solid phase, and target protein (1 μM) was used as the mobile phase. The incubation buffer and cleaning buffer used was 20 mM HEPES, 150 mM NaCl, 2 mM CaCl_2_ (pH 7.4–7.6).

### Self-Assembly of X-polymer Nanoparticles

The X-polymer nanoparticles were assembled in two eppendorf (EP) tubes according to the following system, and the total volume of each tube was 20 μL. EP tube A contained the strands of Del60, 3wj-2Del60, and Del60-3wj. EP tube B contained the strands of T-p and TRS. After the treatment of EP tube A at 95°C 10 min, 4°C 10 min, 22°C 20 min, 47°C 20 min, and 73°C 20 min, the treatment of EP tube B at 85°C 10 min and 4°C 10 min, tube A and B were mixed and incubated at 37°C for 1 h. The strands of Del60, 3wj-2Del60, Del60-3wj, TRS, and T-p were mixed at a molar concentration ratio of 4:1:1:1 in PBS buffer. The assembled X-polymer nanoparticles were stored at −70°C.

### T Cell Proliferation Assay

Spleen tissues of 6- to 8-week-old BALB/c mice (Beijing Vital River Laboratory Animal Technology) were obtained and ground, filtered on a 200 mesh cell sieve, and centrifuged with a horizontal rotor at 650× *g* for 30 min by adding Lymphocyte separation medium (Dakewe Biotech, 7211011). The lymphocyte layer was purified with a nylon hair column (Polyscience, 21759-1) or a mouse CD4/CD8 T cell isolation kit (Biolegend, 480005/480007) and added to 1640 medium for subsequent experiments. T cells were then stained with CFDA-SE dyes (Invitrogen, C34554). T cells (1.6∗10ˆ6/mL) were cultured in 1640 complete medium in a 96-well plate and then incubated with 0.025 μg/well of anti-CD3 antibodies (Biolegend, 100313), 0.5 μg/well of anti-CD28 antibodies (Biolegend, 102111), or 100 pmol/well of aptamers for 72 h. The T cells were detected by flow cytometry. In order to differentiate CD4^+^ and CD8^+^ T cells, anti-CD4 antibodies (P-phycoerythrin (PE)-anti-CD4 antibody, Biolegend, 100407) and anti-CD8 antibodies (APC-anti-CD8 antibody, Biolegend, 100711) were stained and analyzed by flow cytometry.

### ELISA

IFN-γ and granzyme B were quantified by ELISA using a mouse IFN-γ ELISA kit (GenStar, C707-02) and a mouse granzyme B ELISA kit (GenStar, C728-02), while IL-2 was quantified by mouse IL-2 ELISA kit (GenStar, C739-02) according to the manufacturer’s instructions.

### Analysis of Blocking T Cell Inhibitory Pathway

T cells were diluted to 1.6∗10ˆ6/mL and seeded in a 96-well plate with 200 μL/well. After stimulating the T cell proliferation with anti-CD3 antibodies (0.025 μg/well) and anti-CD28 antibodies (0.5 μg/well), B7.1 protein (R&D systems, 740-B1-100; 400 ng/well) was added to inhibit T cell proliferation before aptamers (200 pmol/well) or anti-CTLA-4 antibodies (Biolegend, 106204; 1 μg/well) were added separately. Cells were cultured for 72 h. The content of IL-2 in the supernatant of cell culture was detected by ELISA.

### Flow Cytometry Assay

T cells (1.2∗10ˆ4) were incubated with X-polymer nanoparticles (200 pmol) assembled with fluorescein isothiocyanate (FITC)-labeled TRS sequence or FITC-labeled control adaptor in PBS buffer for 30 min, then washed twice with PBS and detected by flow cytometry. APC-antiCD3 antibody (0.5 μg) was used to label T cells.

### Cell Immunofluorescence Assay

For immunofluorescence assay, 2∗10ˆ4 cells were seeded in a 15-mm diameter cell dish and cultured in full media for 12 h. The X-polymer nanoparticles assembled by Cy5-labeled 3WJ-2 Del60 sequence were added to the cells and imaged by confocal. CFDA-SE dye or Hoechst reagent (Beyotime, C1029) was used to stain cell cytoplasm and nuclei, respectively.

### Apoptosis Assay of X-polymer Nanoparticles to Mouse Melanoma B16 Cells *In Vitro*

B16 cells (4∗10ˆ 4/well) stained with CFSE were seeded in a 96-well plate and cultured in full medium for 12 h. After the culture media was removed, T cells (2 × 10ˆ5/well) and aptamers or antibodies were added. After another 48 h of co-culture, the culture media was removed, while cells were trypsinized and resuspended in PBS buffer. The apoptosis rate was analyzed by flow cytometry with Annexin V-PE (Beyotime, C1065).

### Animal Experiment

To detect the activity of X-polymer nanoparticles *in vivo*, C57BL/6J mice (4 weeks old, obtained from Beijing Vital River Laboratory Animal Technology) were injected with 4∗10ˆ6 B16 cells into left subscapular tissues. The aptamers (400 pmol per mouse) or anti-CD28 antibodies plus anti-CTLA-4 antibodies (anti-CD28 antibody, 4 μg per mouse; anti-CTLA-4 antibody, 8 μg per mouse) were simultaneously administrated into subcutaneous tissues next to the injection site. The total number of times of injections was four. The first and second injections were given every 3 days, while the other injections were given every 2 days, and the dose of the first injection was 400 pmol per mouse. On the ninth day, most of the mice grew tumors. There were obvious protrusions under the left scapula of the mice. The hardening and size of red beans could be clearly felt by the touch of the hand. On the 11th day, the growth of the tumors was obvious. On the 13th day, when the growth of tumors in the control group was close to 1 cm^3^, the tumors were removed for measurement. Tumor volumes were calculated as 0.5 × a (length) × b (width)^2^.

### Statistical Analysis

Data was analyzed by using GraphPad Prism 5.0 software (GraphPad, San Diego, CA, USA). For ELISA assay and T cell proliferation assay, the standard deviation (± SD) was obtained for the three parallel groups of data, and the experiments were repeated three times. We used one-way ANOVA statistics to compare the difference between more than two groups. p < 0.05 was considered statistically significant.

## Author Contributions

N.S., S.L., and P.Z. designed the study. N.S. and C.B. wrote the manuscript. C.B., S.G., and S.H. contributed to carrying out the experiments. X.L., H.L., J.D., A.H., and L.Z. provided technical support.

## Conflicts of Interest

The authors declare no competing interests.

## Supplemental Information

Document S1. Figures S1–S3

Document S2. Article plus Supplemental Information
