# Supplementary material for: Self-Assembled Multivalent Aptamer Nanoparticles with Potential CAR-like Characteristics Could Activate T Cells and Inhibit Melanoma Growth
Source: Mol Ther Oncolytics. 2020 Mar 13;17:9–20. doi: 10.1016/j.omto.2020.03.002 (PMC7139145; doi:10.1016/j.omto.2020.03.002)
Supplement: Document S1. Figures S1–S3 [file mmc1.pdf]

OMTO, Volume *17*

Supplemental Information

Self-Assembled Multivalent Aptamer Nanoparticles with Potential CAR-like Characteristics Could Activate T Cells and Inhibit Melanoma Growth

Chenjun Bai, Shanshan Gao, Sai Hu, Xuemei Liu, Hui Li, Jie Dong, Aixue Huang, Lingling Zhu, Pingkun Zhou, Shaohua Li, and Ningsheng Shao

Supplementary Data

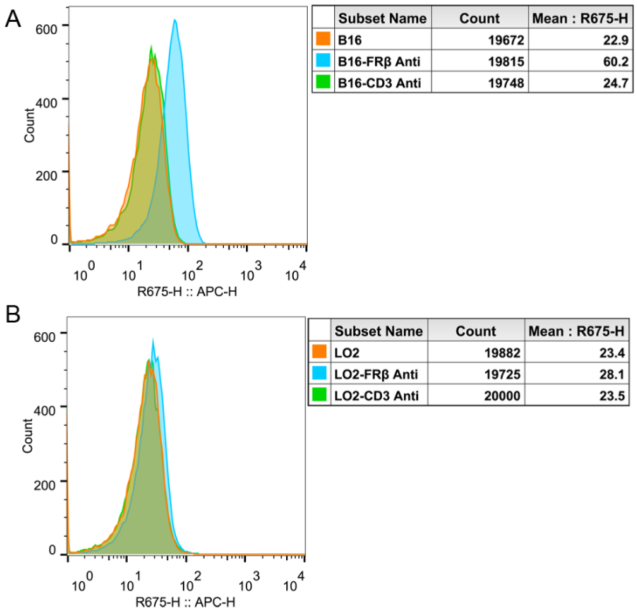

	Subset Name	Count	Mean : R675-H
	B16	19672	22.9
	B16-FRβ Anti	19815	60.2
	B16-CD3 Anti	19748	24.7

	Subset Name	Count	Mean : R675-H
	LO2	19882	23.4
	LO2-FRβ Anti	19725	28.1
	LO2-CD3 Anti	20000	23.5

Figure S1. Detection of folate receptor on B16 cells surface. We used flow cytometry to detect the expression of folate receptor on the surface of B16 cells and LO2 cells (Negative control of folate receptor expression). (A) B16 cells with anti-Folate Receptor β (FR-β) antibodies (Biolegend, 153305) or Anti-CD3 antibodies (negative control). (B) LO2 cells with anti-Folate Receptor β (FR-β) antibodies (Biolegend, 391705) or Anti-CD3 antibodies (negative control).

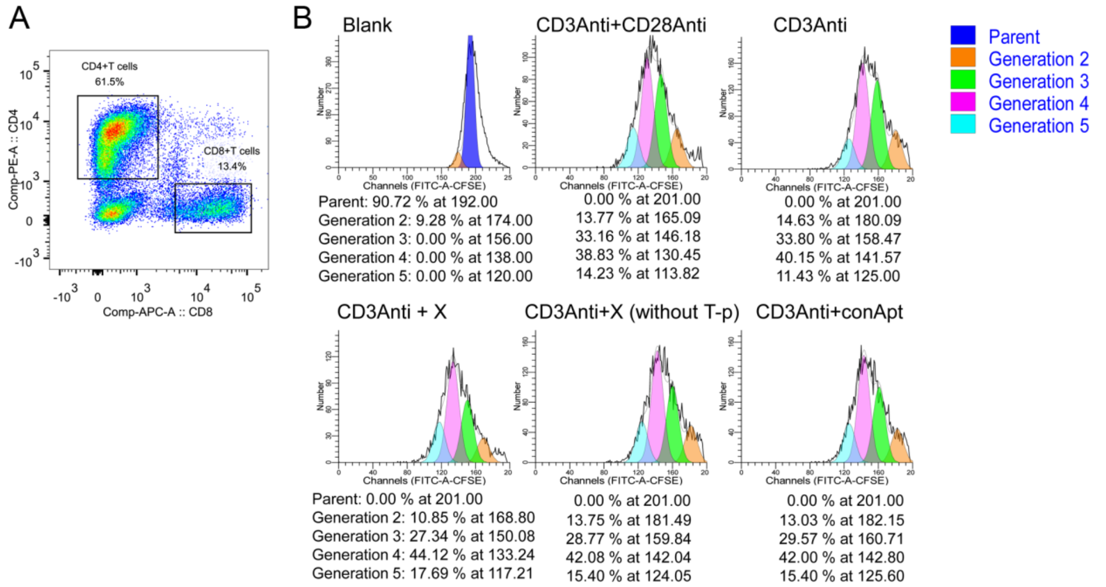

Figure S2. X-Polymer nanoparticles promoted mouse T cell proliferation in vitro. (A) T cell population was treated with PE-labeled Anti-CD4 antibody and APC-labeled Anti-CD8 antibody. (B)Flow cytometry analysis of the CD8+ T cells. T cells were labeled with CFSE, the first activation signal was provided by Anti-CD3 antibodies, and the second signal by Anti-CD28 antibodies or X-polymer nanoparticles.

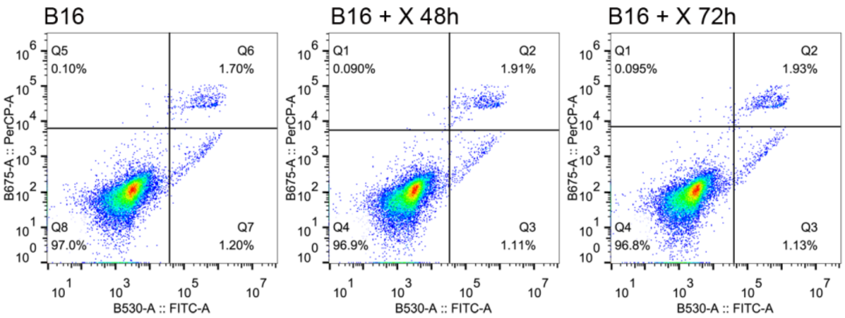

Figure S3. The apoptosis rate of B16 cells co-cultured with X-Polymer nanoparticles. B16 cells (5*10^ 4/well) were seeded in a 96-well plate with X-Polymer nanoparticles (200 pmol/ well) in DMEM full medium for 72 hours. After 48 hours and 72 hours of co-culture, the culture media was removed while cells were trypsinized and resuspended in PBS buffer. The apoptosis rate was analyzed by flow cytometry with Annexin V-FITC and PI (Propidium iodide) dye solution (DOJINDO, AD10).
